# Genetic Diversity, Population Structure, and Environmental Influences in *Nepeta glomerulosa* : Implications for Conservation in the Irano‐Turanian Region

**DOI:** 10.1002/ece3.73294

**Published:** 2026-04-29

**Authors:** Sahar Karami, Hamid Ejtehadi, Jamil Vaezi, Hamid Moazzeni

**Affiliations:** ^1^ Quantitative Plant Ecology and Biodiversity Research Lab, Department of Biology, Faculty of Science Ferdowsi University of Mashhad Mashhad Iran; ^2^ FUMH Herbarium, Department of Biology, Faculty of Science Ferdowsi University of Mashhad Mashhad Iran

**Keywords:** Alborz Mountain range, environmental factors, genetic diversity, Irano‐Turanian region, population structure

## Abstract

This study provides the first comprehensive assessment of the genetic diversity, population structure, and evolutionary history of *Nepeta glomerulosa*, a semi‐endemic medicinal plant of the Irano‐Turanian region with numerous therapeutic uses. Using sequence‐related amplified polymorphism (SRAP) markers, we analyzed 188 accessions from 18 populations across Iran's major mountain ranges: Alborz, Zagros, and Khorassan‐Kopet Dagh. A total of 251 bands were amplified, of which 99.20% (249) were polymorphic. The species exhibited moderate genetic diversity (Nei's gene diversity index, He = 0.2605), with the highest diversity observed in the Alborz range, suggesting it may be the center of origin. Genetic differentiation among populations (Gst = 0.4199) was substantial, with analysis of molecular variance (AMOVA) revealing that 30.17% of the total variation was attributable to differences among populations, while 69.83% was due to variation within populations. Clustering analyses using UPGMA and principal coordinate analysis (PCoA) classified the accessions into two primary clusters, whereas Bayesian STRUCTURE analysis identified three distinct genetic clusters with significant admixture, particularly in populations from the Zagros Mountains. This pattern indicates that the Zagros range may function as an important zone of genetic exchange between northern and southeastern populations. Spatial and environmental variables, including geographic distance, altitude, and edaphic conditions, significantly contributed to the observed genetic structure. Field observations further indicate that *N. glomerulosa* populations are threatened by overgrazing, habitat degradation, and unsustainable harvesting. Considering its restricted distribution and small population sizes, targeted conservation actions are urgently required, especially in vulnerable mountainous regions.

## Graphical abstract

*Nepeta* *glomerulosa* shows moderate genetic diversity with three main clusters and significant admixture, highest in the Alborz Mountains, suggesting it may be the center of origin. High genetic differentiation (Gst = 0.4199) points to limited gene flow and possible self‐pollination, with geographic isolation shaping population structure. Genetic and morphological variation is strongly influenced by altitude, soil conditions, and geographic distance, confirmed by Mantel tests showing isolation by environment and distance. Due to habitat loss, overgrazing, and overharvesting, *N*. *glomerulosa* urgently requires conservation strategies including protected areas, sustainable use, and ex‐situ efforts.

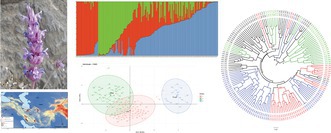

## 1 Introduction

**FIGURE 1:**
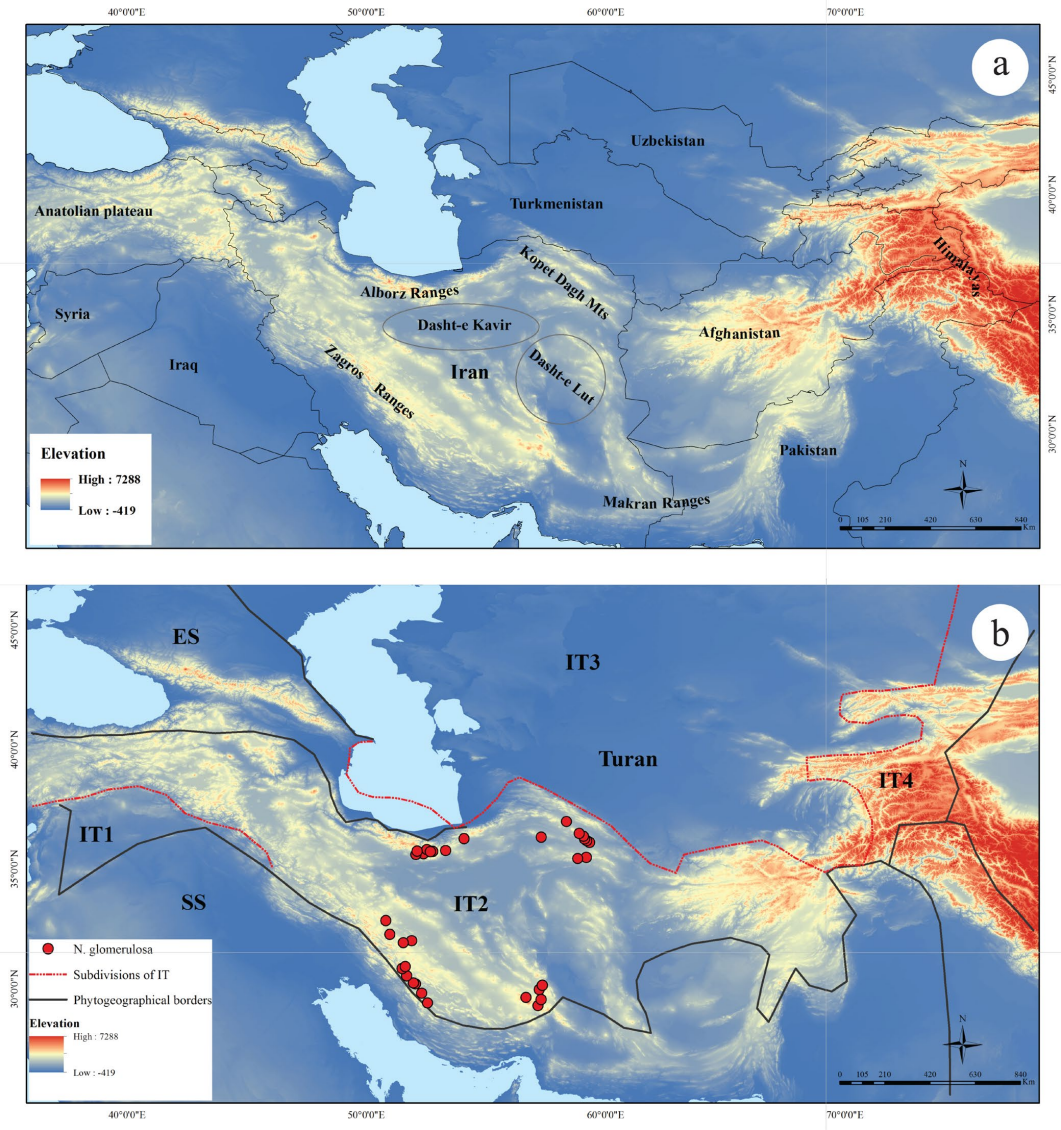
The study area and distribution of *Nepeta glomerulosa* in Iran: (a) Topographic map of mountain areas across Iran, (b) ES, Euro‐Siberian region; IT, Irano–Turanian region; SS, Saharo‐Sindian region.

Endemism refers to the restriction of a species to a single, well‐defined geographic area, often resulting from historical, ecological, and evolutionary processes (Anderson 1994; Noroozi et al. 2019). Iran, a vast country in Southwest Asia (Figure 1), harbors a remarkable diversity of landscapes, characterized by extensive arid‐to‐semiarid mountain systems, intermontane basins, and plateaus. This pronounced topographic and climatic heterogeneity has played a central role in promoting species diversification and the emergence of endemic taxa. Iran supports an exceptionally rich biota, including more than 8000 vascular plant taxa, approximately 30% of which are endemic (Khalvati et al. 2025; Noroozi et al. 2016, 2018). In addition, the country hosts over 1000 species of mainland vertebrates (Firouz 2005), further underscoring its significance as a major center of biodiversity and endemism in the Irano‐Turanian region.

*Nepeta* L. is among the largest genera within the Lamiaceae family, encompassing more than 200 species. The centers of diversity for this genus are situated in the western Himalayas, across Eurasia, and southwestern Asia (Budantsev 1993; Harley et al. 2004; Sharma et al. 2021). Due to their numerous therapeutic properties, such as diuretic, expectorant, antispasmodic, and antiseptic, *Nepeta* species have garnered interest from the pharmaceutical industry (Amirmohammadi et al. 2019; Talebi et al. 2022).

**FIGURE 2:**
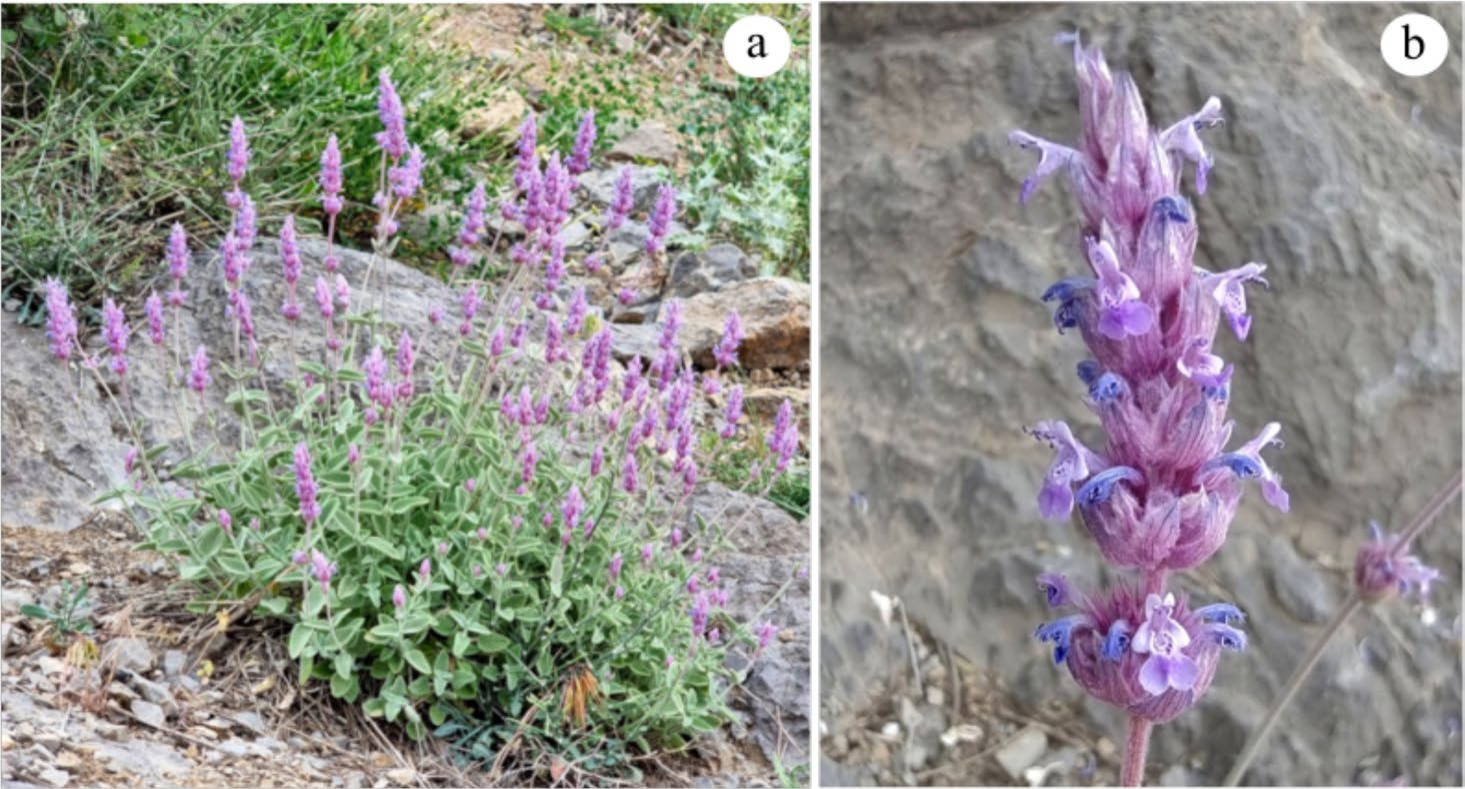
*Nepeta glomerulosa*; (a): life form; (b): morphology of flower (Photos by Ramiar Majidi).

*Nepeta glomerulosa* Boiss. is a semi‐endemic species traditionally used in the treatment of various skin and gastrointestinal disorders, pneumonia, and itching (Amirmohammadi et al. 2019; Talebi et al. 2018). Morphologically, the species is a perennial herb with a woody base, densely covered with a white tomentose‐lanate indumentum, and bearing sessile glandular trichomes. Leaves are ovate with prominent, reticulate venation and dentate margins. Flowers are purple, arranged in a spicate inflorescence; each flower is subtended by a bract, white to purplish in color. Calyx equaling the bract in length, 5‐nerved, with unequal teeth, the lower teeth usually longer than the upper ones. Fruit is an oblong‐rectangular nutlet (Jamzad 2012; Rechinger 1982). *Nepeta glomerulosa* can be found at elevations ranging from 1500 to 4000 m in the Alborz, Zagros, and Khorassan–Kopet Dagh of Iran, as well as in the Paropamisus Mountains of Afghanistan (Bakhshi et al. 2021; Rechinger 1982; Figure 1). This species grows on rocky and gravelly slopes, often co‐occurring with plant communities such as *Astragalus* L., *Artemisia* L., *Amygdalus* L., and *Pistacia* L. It is also found in dry rivers and springs within the Irano–Turanian biogeographical region (Karami et al. 2022). It exhibits considerable morphological diversity, showing variability in vegetative traits (e.g., plant height) and reproductive traits (e.g., bract color and size, calyx dimensions, flower cycle spacing, and inflorescence form). Furthermore, inflorescence morphology undergoes significant transformations during the plant's life cycle, particularly during the transition phases of flowering initiation and termination (Bakhshi et al. 2021; Figure 2). *Nepeta glomerulosa* consists of four subspecies, three of which have overlapping distributions, making them difficult to distinguish. These subspecies are endemic to Iran. They include: (1) *N. stapfiana*, found in western and central Iran; (2) *N. glomerulosa*, present in central regions; (3) *N. carmanica*, located in central, western, southeastern, and eastern Iran (Bakhshi et al. 2021; Rechinger 1982). The fourth subspecies is *N. ghorana*, identified as a distinct subspecies of *N. glomerulosa* in Ghorat, southwestern Afghanistan (Govaerts 2009).

Population genetic structure refers to assessing genetic diversity across space and time, which can yield insights into species distribution, species delimitation, mating behavior, and population boundaries (Janes and Batista 2016; Wang, Dai, et al. 2023; Wang, Jiang, et al. 2023). Numerous studies have been conducted on the genetic structure of populations within the Lamiaceae family, including: *Elsholtzia stauntonii* Benth (Zhang et al. 2022), *Sideritis gulendamii* H. Duman & Karaveliogullari (Yıldırım et al. 2024), *Micromeria* Benth (Puppo et al. 2016), and *Nepeta* (Talebi et al. 2022). Molecular genetic markers serve as a reliable method for genetic analysis at the plant population level (Szabo et al. 2021; Wang, Dai, et al. 2023). Sequence‐related amplified polymorphism (SRAP) is a type of molecular marker technology based on polymerase chain reaction, first introduced by Li and Quiros (2001). SRAP marker is reliable, simple, genome‐specific, easily detected, highly polymorphic (Huang et al. 2012; Ma et al. 2015; Robarts and Wolfe 2014), and very effective for assessing genetic diversity (Al Shaye et al. 2018; Aneja et al. 2013). These reasons have led to the widespread use of SRAP markers in the population genetic analysis of various plant species (Wang et al. 2019; Wang, Dai, et al. 2023).

Although *N. glomerulosa* is a semi‐endemic medicinal species and is facing taxonomic (e.g., morphological similarity with related taxa and limited molecular data) and ecological challenges (e.g., restricted distribution, habitat fragmentation, overharvesting, and in some populations, diverse forms suggesting the influence of local ecological conditions on morphological variation), no comprehensive information is available on its population genetic structure. Previous studies have primarily focused on its hypnotic effects (Hosseini et al. 2016), essential oils (Emamia et al. 2017; Javidnia et al. 2008), and the impact of climate change on its distribution (Karami et al. 2022). In this study, we employed SRAP to evaluate the genetic diversity and population structure of *Nepeta glomerulosa*. Additionally, we investigated potential factors contributing to population differentiation, including geographic distance, as well as edaphic and topographic variables.

## 2 Materials and Methods

### 2.1 Sample Information

This study involved collecting 188 individuals for morphological analyses and 134 for molecular analyses from 18 wild populations of *N. glomerulosa* across its known distribution range. Populations were selected to represent the full geographic, ecological, and environmental variability of the species, including differences in altitude, habitat type, and climatic conditions. Within each population, individuals were sampled at sufficient spatial intervals to minimize the likelihood of collecting closely related plants, and only healthy, mature individuals were selected.

Field collections were carried out during the main growing season (May–June), when diagnostic morphological characters were fully developed. Leaf material for molecular analyses was collected in the field and preserved immediately, while voucher specimens were prepared and deposited in the Ferdowsi University of Mashhad Herbarium (FUMH).

Unfortunately, we were unable to conduct fieldwork in the Parapamis Mountains in southwestern Afghanistan, where the subspecies *N. glomerulosa* subsp. *ghorana* is found. Taxa sampled, voucher information, and population ID are presented in Table 1.

**TABLE 1:** The geographic information of 18 *N. glomerulosa* populations in the Iran Mountains.

Pop ID	Pop number	Latitude	Longitude	Altitude	Number of individuals	Administrative area
MB	1	36°7′36.99″	59°19′50.14″	2317	17	Razavi Khorasan, Binalud, Moghan
ZGD	2	36°22′24.48″	59° 4′29.86″	2045	18	Razavi Khorasan, Golmakan
SA	3	36°20′48.58″	57°18′45.58″	1518	5	Razavi Khorasan, Saruq
QA	4	36°59′37.05″	58°21′37.09″	1900	6	Razavi Khorasan, Quchan
TMK	5	35°26′53.07″	58°50′16.16″	1750	10	Razavi Khorasan, Torbat‐e Heydarieh
CHD	6	36°16′41.08″	54° 4′52.83″	1500	5	Semnan, Damghan, Cheshmeh Ali
DF	7	35°38′42.19″	52°23′2.28″	2130	23	Tehran, Saidabad
QS	8	35°36′37.10″	52° 3′44.77″	2050	6	Tehran, Absard
RLH	9	29°33′43.08″	57°17′55.63″	2838	15	Kerman, Kūh‐e Hazār
BJ	10	29°58′50.81″	57°13′37.99″	2350	10	Kerman, Jupar
SHQ	11	29°49′27.66″	52°18′25.99″	2113	5	Shiraz, Qalat
SBR	12	30°12′26.58″	52° 2′54.29″	2200	11	Fars, Sepidan, Bereshneh
SHS	13	29°25′0.26″	52°33′22.39″	1550	5	Fars, Shiraz, Sabzpooshan
SMO	14	30°33′12.17″	51°40′45.46″	2000	5	Sepidan, Emamzade Mohammad
YSD	15	30°51′51.05″	51°29′48.52″	2490	14	Yasuj, Dena
SHF	16	32°16′53.35ign="center">30°33′12.17″	51°40′45.46″	2000	5	Sepidan, Emamzade Mohammad
YSD	15	30°51′51.05″	51°29′48.52″	2490	14	Yasuj, Dena
SHF	16	32°16′53.35″	50°58′37.76″	2152	5	Shahrekord, Farrokh Shahr
BDR	17	32° 0′18.80″	51°53′9.50″	1850	13	Isfahan, Shahreza
CHM	18	32°51′10.22″	50°49′0.36″	2202	9	Isfahan, Cheshmeh Morghab

### 2.2 Molecular Study

A total of 188 plants from 18 populations (5–23 specimens per population) were used to analyze genetic diversity and population structure, as detailed in Table 1. Fresh leaves were collected from each population and dried in silica gel.

#### 2.2.1 Genomic DNA Extraction and SRAP Amplification

We utilized a modified CTAB protocol for DNA extraction from the Lamiaceae family (Talebi et al. 2022; Zagorcheva et al. 2020) then employed nanograph (by NanoDrop 2000c spectrophotometer) and 1.5% gel to assess the quantity and quality of the extracted DNA. A total of 36 different SRAP primer pairs were evaluated, of which 12 primer pairs were selected based on the efficiency of the PCR reaction and the number of bands (Table 2). The procedure for SRAP‐PCR amplification was adapted from methods previously used for other Lamiaceae species, with modifications (Aghaei et al. 2017; Hmissi et al. 2024). The following cycling parameters were used for amplification: Initial denaturation at 94°C for 3 min, followed by 5 cycles of denaturation at 94°C for 60 s, annealing at 35°C for 60 s, and extension at 72°C for 60 s. Then, for the subsequent 35 cycles, an initial denaturation at 94°C for 60 s, annealing at 50°C for 60 s, and extension at 72°C for 60 s was performed, followed by a final extension step at 72°C for 10 min. The amplified products were stored at 4°C. Following amplification, PCR products were separated by electrophoresis on a 1.5% agarose gel.

**TABLE 2:** Results of SRAP amplification with 12 primer combinations.

Primer ID	Sequence	Size of loci (bp)	Number of loci	Number of polymorphic loci	Percentage of polymorphic loci (%)	Polymorphic information content
F8/R8	TGAGTCCAAACCGGACT/GACTGCGTACGAATTCAC	50–1600	24	24	100%	0.23
F8/R9	TGAGTCCAAACCGGACT/GACTGCGTACGAATTCAG	50–1700	24	24	100%	0.27
F8/R10	TGAGTCCAAACCGGACT/GACTGCGTACGAATTCAT	50–1600	21	21	100%	0.28
F8/R11	TGAGTCCAAACCGGACT/GACTGCGTACGAATTCTA	50–1700	21	21	100%	0.28
F9/R8	TGAGTCCAAACCGGAGG/GACTGCGTACGAATTCAC	50–1300	18	18	100%	0.20
F9/R9	TGAGTCCAAACCGGAGG/GACTGCGTACGAATTCAG	50–1500	17	17	100%	0.21
F9/R10	TGAGTCCAAACCGGAGG/GACTGCGTACGAATTCAT	50–1500	18	17	94.44%	0.24
F9/R11	TGAGTCCAAACCGGAGG/GACTGCGTACGAATTCTA	50–1500	23	23	100%	0.28
F9/R12	TGAGTCCAAACCGGAGG/GACTGCGTACGAATTCTC	50–1700	25	25	100%	0.33
F9/R13	TGAGTCCAAACCGGAGG/GACTGCGTACGAATTCTG	50–1600	21	21	100%	0.22
F9/R20	TGAGTCCAAACCGGAGG/GACACCGTACGAATTGAC	50–1700	22	21	95.45%	0.25
F9/R21	TGAGTCCAAACCGGAGG/GACACCGTACGAATTTGA	50–1600	17	17	100%	0.33

#### 2.2.2 Data Statistics and Analysis

Amplified fragments were scored based on the presence (1) or absence (0) of homologous bands, with only reproducible bands included in the analysis. The band data were then transformed into a binary matrix for each primer combination (Robarts and Wolfe 2014).

We used POPGENE version 1.32 (Yeh et al. 1999) to compare amplification results across various primer combinations, including the number of loci, fragment sizes, and the percentage of polymorphic sites. The Polymorphic Information Content (PIC) was calculated using the formula PIC = 1 − *p* ^2^ − *q* ^2^ (*p* represents the ratio of bands and *q* denotes the ratio of no bands) (Sharma et al. 2016). Genetic indices, including sample size, number of alleles (Na), effective number of alleles (Ne) (Kimura and Crow 1964), Nei's gene diversity (He) (Nei 1973), and Shannon's Information index (I) (Lewontin 1972), as well as the number and percentage of polymorphic loci, were computed to estimate genetic diversity within different populations of *N. glomerulosa*. The total genetic diversity (Ht), diversity within a population (Hs), and genetic differentiation coefficient (Gst) were also determined. Gene flow (Nm) was calculated using the formula: Nm = 0.5 (1—Gst)/Gst.

Principal coordinate analysis (PCoA) of the 18 populations was conducted using GenAlex version 6.5 (Peakall and Smouse 2006). Molecular analysis of variance (AMOVA) was employed in Arlequin version 3.5.2.2 (Excoffier and Lischer 2010) to assess genetic variation within and among populations.

The Phylip version 3.6981 software package (Felsenstein 1993) was applied to calculate the genetic distance matrix. The unweighted pair‐group method of arithmetic averages (UPGMA) (Sneath and Sokal 1963) tree was constructed based on the genetic distance matrix using the Phylip software package.

To survey the population genetic structure of the sampled accessions of *N*. *glomerulosa*, a common‐line version of STRUCTURE 2.3 was used to perform a Bayesian population assignment analysis (Pritchard et al. 2000). Values of *K* = 1–12 were used separately to run STRUCTURE. The initial burn‐in period for each run was set to 100,000, with 100,000 Monte Carlo Markov Chain iterations. The pophelper package (Francis 2017) as implemented in R was used to find the optimal K value. LibreOffice in Ubuntu 22.0.4 customized the graphic representations of the accessions. The number of subpopulations was calculated using the ΔK method (Evanno et al. 2005). According to the probability of membership (*Q* value) of > 70% and < 70%, the *N*. *glomerulosa* accessions were classified as either a subpopulation or a mixed subpopulation, respectively (Liu et al. 2013).

ClustVis version 2.0, a web‐based tool for the visualization of clustering in multivariate data (BETA [https://biit.cs.ut.ee/clustvis]), was utilized to generate a heat map illustrating the correlations and distinctions of SRAP loci across various populations, as well as to perform Principal Component Analysis (PCA).

### 2.3 Morphological Study

For the morphological study, a total of 134 plants from 18 populations (3–8 specimens) were sampled and identified using the Flora of Iran (Jamzad 2012) and Flora Iranica (Rechinger 1982). In the first stage, forty morphological traits were evaluated (Data S1). We used IBM SPSS Statistics software (version 26) to obtain correlations. For quantitative data, we used Pearson's correlation; for qualitative data, we used Spearman's correlation. Then, for traits that were not correlated and normal, we used ANOVA; for traits that were not normal or qualitative, we used the Kruskal‐Wallis test between groups; and traits with significant differences were selected for the next stage. Finally, five main traits (plant height (ph), bract length (bl), calyx length (cl), and corolla length (ctl)) along with bract color (bc) were selected for the final analysis (Data S2). Measurements were taken using a ruler, a digital caliper, a scrubber, and an optical microscope.

#### 2.3.1 Morphological Analyses

PCA was conducted in R version 4.4.1 on morphometric data from 134 individuals across 18 populations, analyzing four quantitative traits (plant height (ph), bract length (bl), calyx length (cl), and corolla length (ctl) along with bract color (bc)) as a qualitative trait.

The analysis was conducted using PAST version 4.17 (Hammer et al. 2001) to perform a redundancy analysis (RDA) to model linear relationships among environmental predictors, genomic variation, and morphometric variation.

### 2.4 Environmental Data

During sampling, both geographic and edaphic features were recorded for each population across different regions of Iran. Geographic variables, including longitude, latitude, altitude, slope, and aspect, were measured in situ using a handheld GPS device (accuracy ±3–5 m), clinometer, and compass. These variables were selected because they capture spatial heterogeneity and potential barriers to gene flow. Soil samples were collected from the root zone (5–30 cm depth) to assess edaphic characteristics, including nitrogen (N), phosphorus (P), potassium (K), carbon (C), calcium (Ca^2+^), pH, and electrical conductivity (EC), which directly affect plant nutrition, growth, and local adaptation. Standard laboratory protocols were followed for soil analyses: total nitrogen was measured using the Kjeldahl method, potassium using flame photometry, soil organic carbon using the Walkley‐Black method, EC (electrical conductivity) using an EC meter, Phosphorus using the Olsen method, and pH using a Jenway pH Meter. Redundancy analysis (RDA) and Mantel test for environmental data were performed using Past software (version 3.14) and R (version 4.4.1), respectively.

## 3 Results

### 3.1 Molecular Result

#### 3.1.1 Polymorphism Analysis of SRAP Amplified Products

A total of 36 pairs of SRAP primer combinations were screened. The amplification bands of the 12 pairs were clear and evenly distributed and were used to estimate the genetic diversity of *N. glomerulosa*. These SRAP primer combinations amplified 251 loci (249 polymorphic loci, 99.20%) with a size range of 50–1700 bp. The primer combination F9/R10 (94.44%) and F9R20 (95.45%) showed the lowest polymorphism, whereas the highest polymorphism (100%) was detected for the primer combinations F8/R8, F8R9, F8R10, F8R11, F9R8, F9/R9, F9R11, F9R12, F913, and F9R21. The range of polymorphism information content (PIC) was from 0.20 to 0.33 (Table 2).

#### 3.1.2 Genetic Diversity and Genetic Structure Analyses

Results for the genetic diversity parameters using the POPGENE program are displayed in Table 3. The Na, Ne, H, and I values at the species level are 1.992, 1.4286, 0.2605, and 0.4048, respectively. At the population level, presuming a Hardy–Weinberg equilibrium, population DF (Na = 1.7211, Ne = 1.3608, H = 0.2177, I = 0.335) had the highest genetic diversity, followed by population SA (Na = 1.1594, Ne = 1.1275, H = 0.0708, I = 0.1014), which had the lowest genetic diversity. The mean Ht, Hs, and Gst values are 0.2663, 0.1545, and 0.4199, respectively. The Nm value was 0.6906, which was less than 1, indicating restricted gene exchange among populations. The results of the AMOVA demonstrated 30.1698% variance among populations and 69.8301% variance within populations (Table 4).

**TABLE 3:** Genetic diversity within 18 natural populations of *N. glomerulosa*.

Pop ID	Pops	Sample size	Na	Ne	He	I	NPL	PPB (%)
MB	1	14	1.5299	1.2731	0.1658	0.2547	133	52.99%
ZGD	2	16	1.5976	1.3228	0.1933	0.2944	150	59.76%
SA	3	3	1.1594	1.1275	0.0708	0.1014	40	15.94%
QA	4	5	1.3068	1.213	0.1223	0.1795	77	30.68%
TMK	5	9	1.4661	1.2719	0.1613	0.2433	117	46.61%
CHD	6	3	1.1992	1.1594	0.0885	0.1268	50	19.92%
DF	7	23	1.7211	1.3608	0.2177	0.335	181	72.11%
QS	8	5	1.3068	1.1942	0.1156	0.1722	77	30.68%
RLH	9	13	1.5458	1.2702	0.1658	0.2564	137	54.58%
BJ	10	8	1.4661	1.2756	0.1633	0.2459	117	46.61%
SHQ	11	4	1.3347	1.2166	0.1286	0.1908	84	33.47%
SBR	12	9	1.5737	1.3866	0.2201	0.3242	144	57.37%
SHS	13	5	1.3506	1.2292	0.1351	0.2002	88	35.06%
SMD	14	5	1.3108	1.2165	0.1243	0.1823	78	31.08%
YSD	15	12	1.5936	1.3401	0.2013	0.3039	149	59.36%
SHF	16	5	1.3307	1.2181	0.1284	0.19	83	33.07%
BDR	17	12	1.5936	1.3516	0.2061	0.3096	149	59.36%
CHM	18	8	1.4661	1.2957	0.1719	0.2559	117	46.61%
Species level		160	1.992	1.4286	0.2605	0.4048	249	99.20%

*Note:* Na = Observed number of alleles; Ne = Effective number of alleles (Kimura and Crow 1964); He = (Nei's 1973) gene diversity; I = Shannon's Information index (Lewontin 1972).

Abbreviations: NPL, the number of polymorphic loci; PPB, percentage of polymorphism bands.

**TABLE 4:** AMOVA analysis of 18 pops of *N. glomerulosa* with SRAP data.

Source of variation	Sum of squares	Variance components	Percentage variation
Among populations	7130.383	20.52836	30.16984%
Within populations	14769.589	47.51429	69.83016%
Total	21899.972	68.04265	100%

#### 3.1.3 Population Cluster Analysis

**FIGURE 3:**
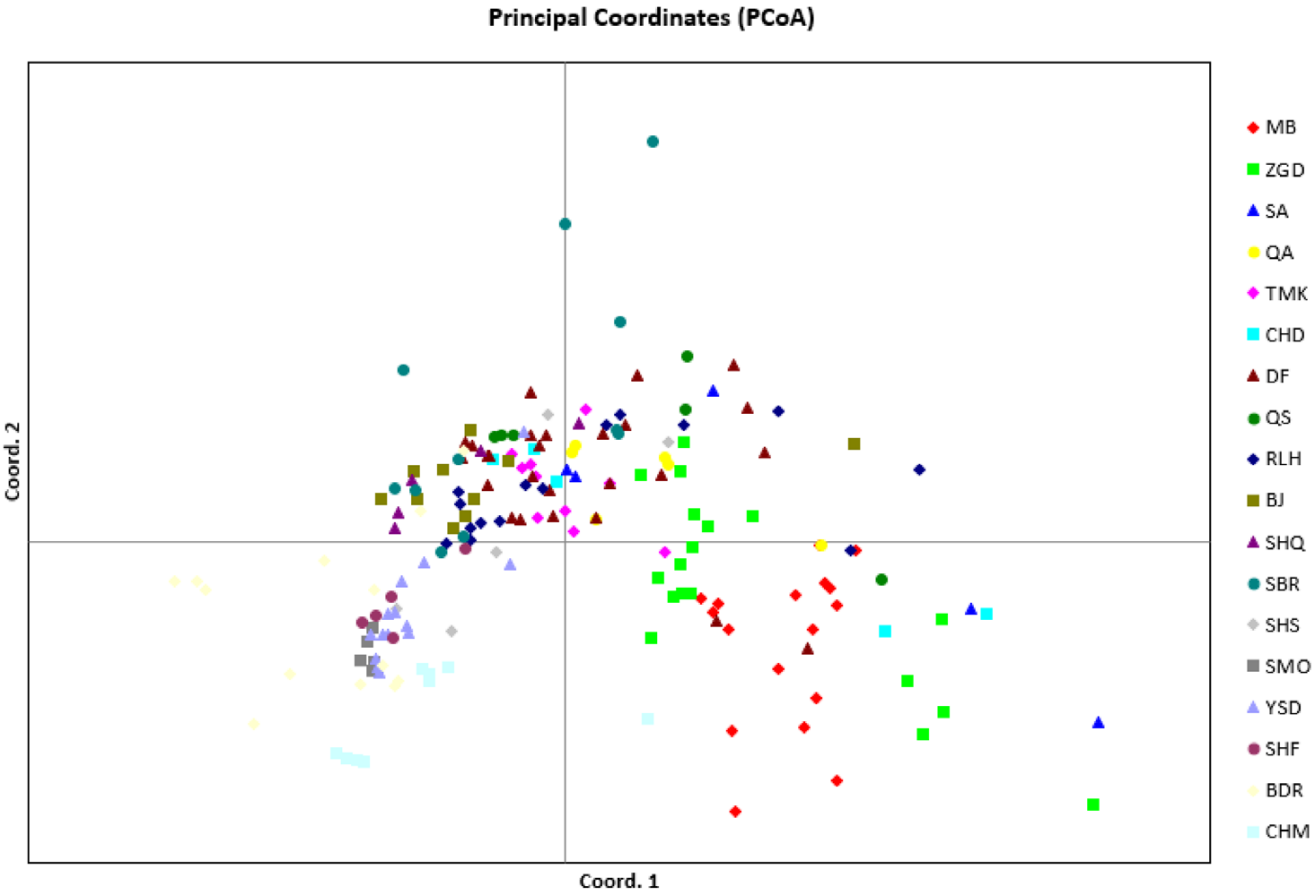
Principal coordinate analysis (PCoA) based on genetic distance illustrates the relationships among the 18 studied populations.

Principal coordinate analysis (PCoA) of 18 *N*. *glomerulosa* populations revealed that these populations were divided into two distinct subgroups (Figure 3). Subgroup I, represented by populations MB, ZGD, SA, QA, and subgroup II, represented by populations RLH, BJ, SHQ, SBR, SHS, SMO, YSD, SHF, BDR, and CHM. The populations CHD and DF were scattered between the two subgroups.

**FIGURE 4:**
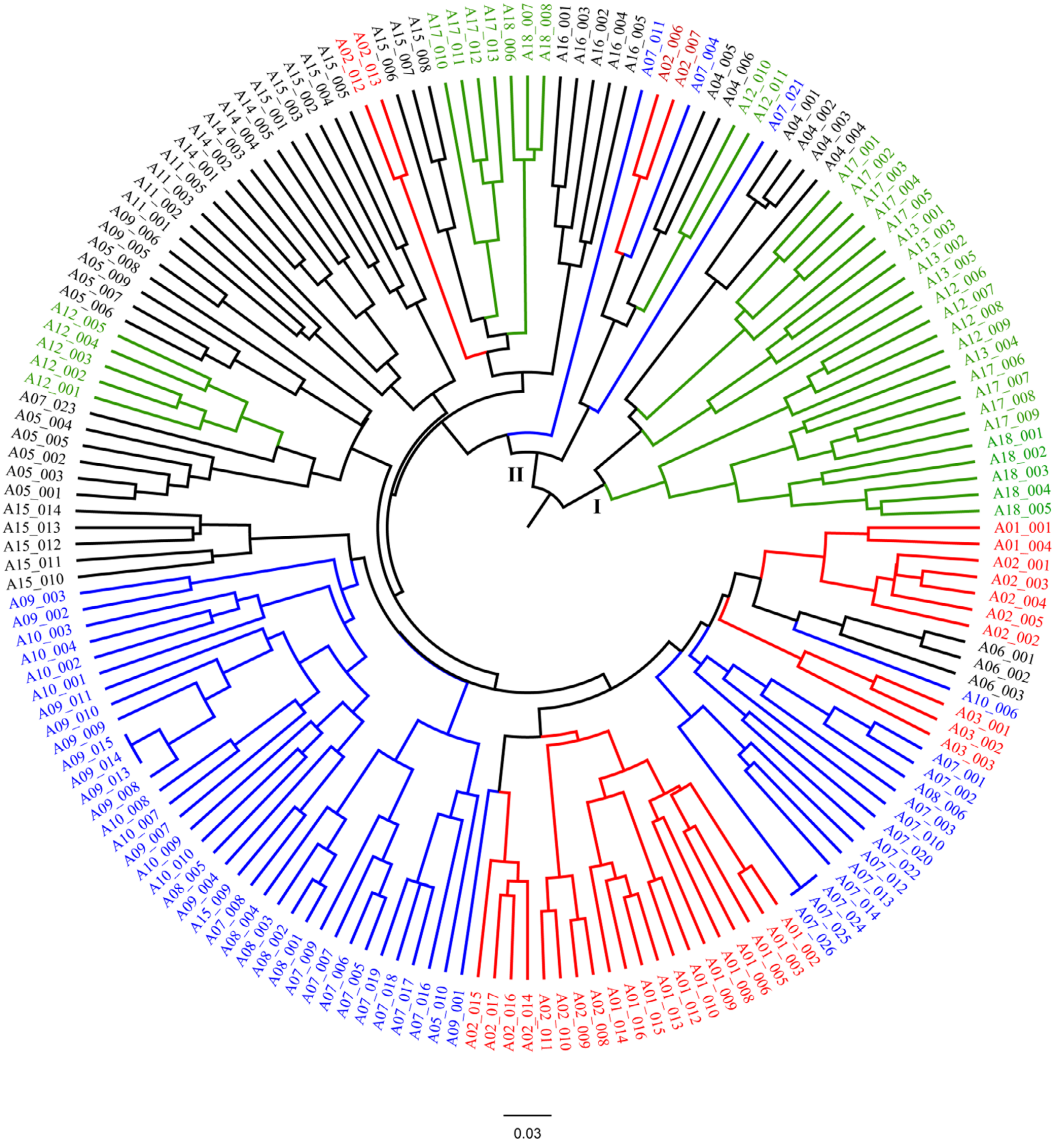
UPGMA dendrogram based on genetic distance values shows the relationship among 188 accessions of *N. glomerulosa*. The different colors in the dendrogram correspond to population structure as identified in the STRUCTURE analysis. Each color represents a certain subgroup (subgroup C1 = red; C2 = green; C3 = blue; The different colors in the dendrogram correspond to population structure as identified in STRUCTURE analysis, and balck color is mixed populations). The population corresponding to each individual is presented in Table 1.

Data generated by SRAP were used to construct a dendrogram using the unweighted pair group method with arithmetic averages (UPGMA) and the Jaccard similarity index. Clustering analyses using UPGMA classified the accessions into two primary clusters (Clades I and II). Clade I comprises five populations (QA, SBR, SHS, BDR, and CHM) from the Zagros Mountain range in central Iran. Clade II includes populations MB, ZGD, SA, TMK, CHD, DF, QS, RLH, BJ, SHQ, SMO, YSD, and SHF, which are distributed across the Kopet Dagh, Alborz, and Zagros Mountain ranges (Figure 4). In both PCoA and UPGMA analyses, accessions from the same or neighboring geographic areas were predominantly grouped into common clusters.

**FIGURE 5:**
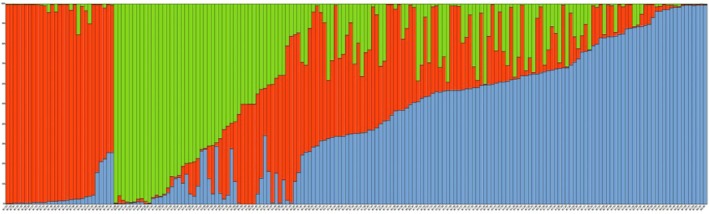
Population structure analysis of 188 *N. glomerulosa* accessions using STRUCTURE (*K* = 3). The *Q* value of each color bar describes the probability of membership fractions of each cluster. When the *K* was 3, there were 3 single‐lineage dominant clusters (red, blue, and green) and the remaining mixed‐lineage cluster.

The population structure of 188 accessions of *N*. *glomerulosa*, obtained from STRUCTURE (Figure 5), is represented using a log mean probability and a Bayesian bar graph to illustrate the clusters and admixture model. Based on the membership fractions, the accessions were categorized into subpopulations and mixed subpopulations. The accessions with membership probability of more than 70% were assigned to clusters (subpopulations), and those with less than 70% fell into mixed subpopulations (Admixture). The STRUCTURE analysis revealed three primary clusters (Figure S1). The three clusters correspond to subpopulations C1 (red), C2 (green), and C3 (blue) (Figure 5). Of 188 accessions, 29 accessions (15.43%) were assigned to subpopulation C1, mainly contained accessions sourced from the Khorassan–Kopet Dagh Mountains (populations MB, ZGD, and SA). The subpopulation C2 includes 28 accessions (14.89%), mostly from the Zagros Mountain range (populations SBR, SHS, *BDR*, and CHM). The subpopulation C3 (blue color) consists of 36 accessions (19.15%) that were mostly from the Alborz Mountain range (populations DF and QS) and Zagros Mountain range (populations RLH and BJ). The remaining 93 accessions (49.46%) appeared to have ancestry from more than one subpopulation, having *Q* values less than 70% (Figure 5).

#### 3.1.4 Heat Map and PCA

Figure S2 shows a heat map illustrating the correlation and differentiation of SRAP loci across various populations. The top dendrogram clusters similar loci, and the left dendrogram groups similar populations. Along the vertical axis, 3 to 5 major clusters can be identified, and the horizontal axis also reveals 3 distinct clusters. Blue areas represent low values (low correlation), red areas indicate high values (high correlation), and white spaces correspond to missing or neutral values. Figure S3 displays the results of Principal Component Analysis (PCA). The x‐axis represents Principal Component 1 (PC1), which explains 21.1% of the total variance, while the y‐axis represents Principal Component 2 (PC2), accounting for 9.7% of the variance. Together, these components provide insights into the primary axes of genetic variation among the populations.

#### 3.1.5 Redundancy Analysis (RDA)

**FIGURE 6:**
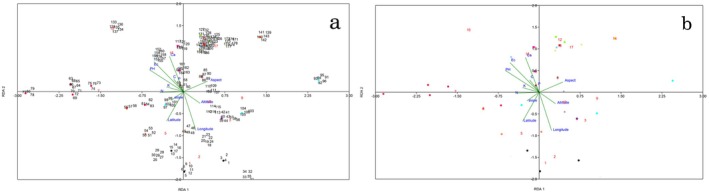
Redundancy Analysis (RDA) based on genetic and environmental data for 18 populations of *N. glomerulosa*; a = RDA based on individuals; b = RDA based on populations.

Redundancy Analysis (RDA) results based on environmental and genetic data are shown in Figure 6. Edaphic factors, including phosphorus (P), carbon (C), calcium (Ca^2+^), electrical conductivity (EC), and pH, contributed to the isolation of populations SHQ, SHS, YSD, and SHF. The factor Nitrogen (N) was associated with the differentiation of population DF, and the slope influenced the isolation of population CHD. Aspect played a role in separating populations QS, SBR, SMO, BDR, and CHM. Altitude was linked to the isolation of populations SA, QA, BLH, and BJ. Geographic factors such as latitude and longitude further differentiated populations TMK and MB/ZGD, respectively.

#### 3.1.6 Mantel Test

The Mantel test results (Data S2), based on Spearman's rank correlation, demonstrated significant correlations between genetic and topographic variables (*R* = 0.07279, *p* < 0.05) as well as between genetic and geographic data (*R* = 0.1872, *p* < 0.01).

### 3.2 Morphological Result

#### 3.2.1 Principal Component Analysis

**FIGURE 7:**
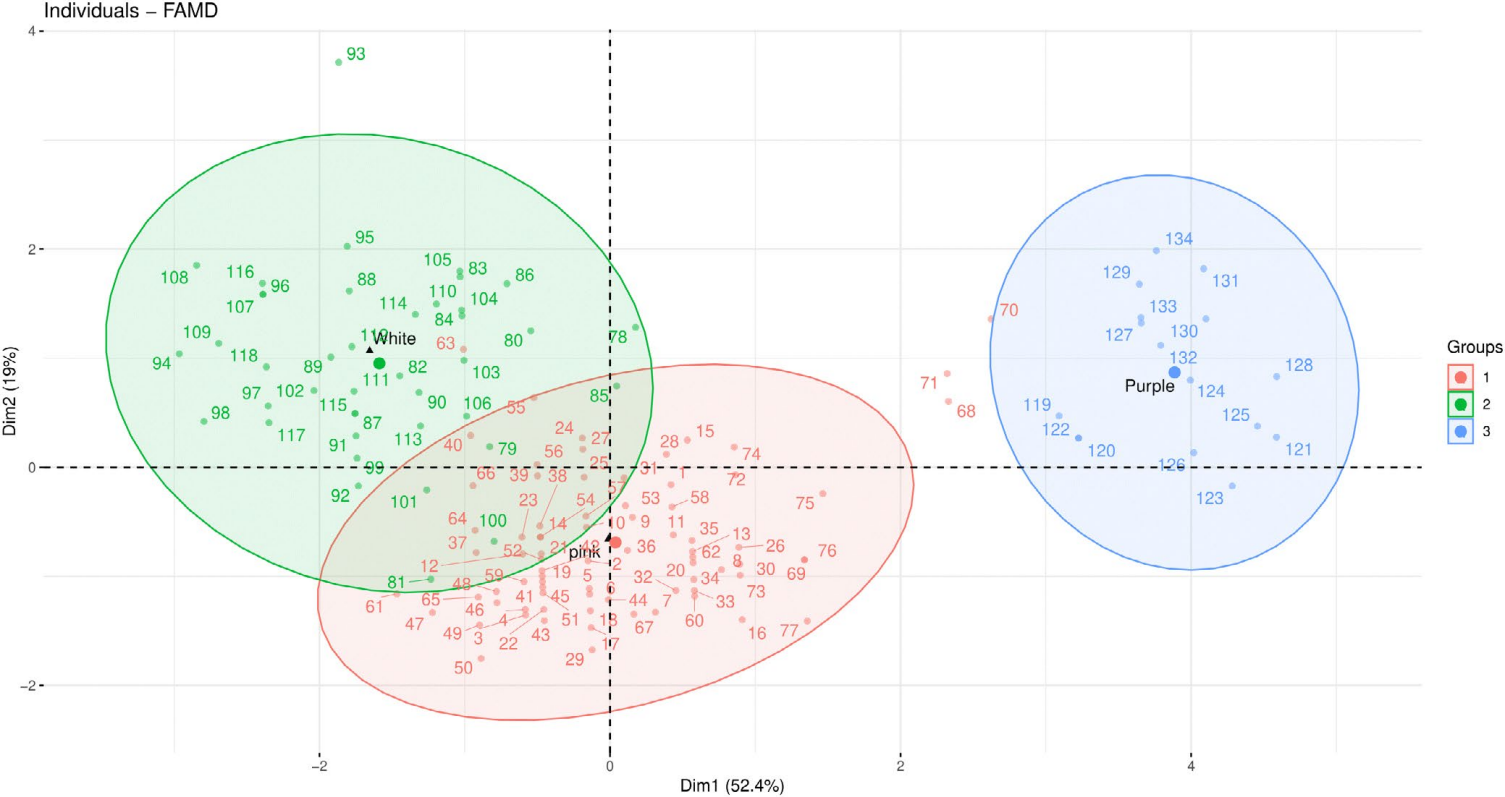
Principal component analysis (PCA) for 18 populations of *N. glomerulosa* based on morphometric data (1 = subsp. *N. carmanica*; 2 = subsp. *N. glomerulosa*; 3 = subsp. *N. stapfiana*).

The Principal Component Analysis (PCA) for 18 populations of *N. glomerulosa* showed that these populations were divided into three distinct subgroups (Figure 7), with two subgroups (subgroup 2; *N. glomerulosa* and subgroup 3; *N. stapfiana*) completely separated. Axis one accounts for 52.4% and axis two accounts for 19%, respectively. In the first axis, the attributes cl, bc, ctl, and bl play the most important role, whereas in the second axis, the attributes bc and ph play the most important role (Figures 7 and S4).

#### 3.2.2 Redundancy Analysis (RDA)

**FIGURE 8:**
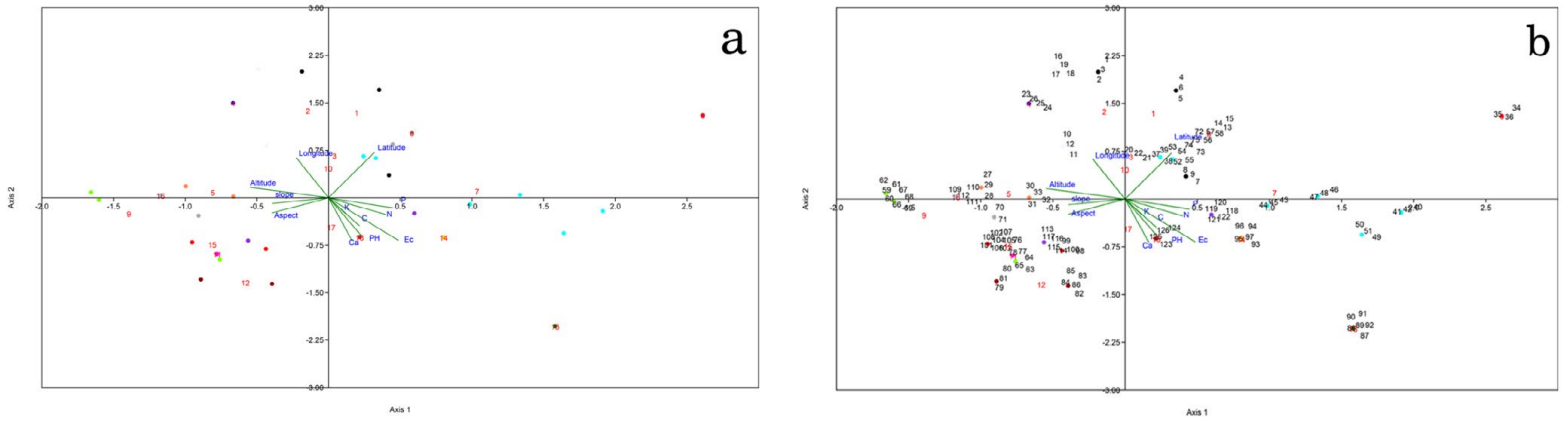
Redundancy Analysis (RDA) based on morphometric and environmental data for 18 populations of *N. glomerulosa*; a = RDA based on populations; b = RDA based on individual.

Figure 8 shows the results of Redundancy Analysis (RDA) based on morphometric and environmental data. Edaphic factors (K, C, Ca^2+^, N, EC, and PH) influenced populations SHS, SMO, BDR, and CHM, leading to their separation from other populations. The factor P caused population DF to be isolated from other populations. Aspect has caused populations SHQ, SBR, and YSD to be isolated from other populations. Altitude and slope have caused populations TMK, RLH, and SHF, latitude has caused populations MB and CHD, and latitude with longitude have caused populations ZGD, SA, QA, and BJ to be isolated from other populations.

#### 3.2.3 Mantel Test

Based on Spearman's rank correlation, the Mantel test results demonstrated a significant correlation between morphometric and geographic data (*R* = 0.0821, *p* < 0.01). However, no correlation was found between the morphometric and topographic data (*R* ^2^ = 0.0001621, *p* > 0.05).

## 4 Discussion

This investigation offers the inaugural molecular and morphological characterization of genetic and phenotypic variation in *Nepeta glomerulosa*, an endemic Lamiaceae species distributed across Iran's Alborz, Zagros, and Khorassan‐Kopet Dagh mountain ranges.

### 4.1 Genetic Diversity of *N. glomerulosa*

Colonization opportunities and limited gene flow at new sites make plant populations isolated, and genetic diversity decreases (Zhang et al. 2019). To date, the population structure and genetic diversity of *N. glomerulosa* have not yet been reported. A total of 251 loci were amplified, of which 249 (99.20%) were polymorphic. Indices such as Na, Ne, He, and I exhibited variability among sample collections and revealed genetic diversity within and among sampled populations. The results from a species perspective showed low genetic diversity in *N*. *glomerulosa*, with a He of 0.2605, which is lower than the average He (0.55) for short‐lived perennials reported in prior genetic studies (Nybom 2004). From a population‐level perspective, the genetic diversity of *N. glomerulosa* in the Alborz Mountain ranges is significantly higher than in the Zagros and Khorassan–Kopet Dagh Mountains. The DF population in the Alborz Mountain range exhibits the highest genetic diversity at 72.11%, while the SA population in Khorassan–Kopet Dagh Mountains has the lowest at 15.94%. The reduced genetic diversity in the northeastern and southern populations may result from limited gene flow from northern populations, due to geographic and ecological isolation across the mountains and valleys (Fetter and Weakley 2019; McGreevy Jr. et al. 2024). In contrast, northern Iran faces no significant geographical barriers, allowing for a centralized population distribution and increased gene flow, thereby enhancing genetic diversity and reducing divergence among these populations (Hosseinian Yousefkhani and Nabizadeh 2025). In addition, the Alborz Mountain Range, which is a possible center of origin for this species, likely contributes to its high genetic diversity.

Multiple factors, including gene flow, selection, mutation, and genetic drift, determine genetic differentiation (Gst) among populations. Gene flow counteracts genetic differentiation (Oh and Oh 2022). Gst indicates the ratio of inter‐population variation to the total genetic variation within a species and serves as a key metric for assessing population genetic differentiation. An increase in Gst signifies a substantial level of differentiation among populations. Gst values greater than 0.25 offer substantial genetic differentiation (Wright 1984). The findings of Nybom (2004) and Hamrick (1987) indicate that species exhibiting a Gst close to 0.5 represent certain self‐pollination (Vilperte et al. 2021; Wang, Dai, et al. 2023; Wang et al. 2014). This observation supports the presence of selfing in *N*. *glomerulosa*, which has a Gst of 0.4199, attributed to the considerable distance separating neighboring populations. Self‐pollination, in the absence of pollinators, can increase population growth and ensure the species' reproduction. This strategy helps gain a competitive advantage in this niche (Sun et al. 2005). However, the poor quality of seeds produced by self‐pollination in the absence of pollinators reduces the population growth rate of *N. glomerulosa* (Law et al. 2010), which could explain its small population size (Karami et al. 2022).

### 4.2 Genetic Structure of *N. glomerulosa*

The genetic structure of *N. glomerulosa* suggests relatively weak differentiation across its distribution range, despite its occurrence in distinct mountain systems. The lack of strong regional genetic separation indicates extensive gene flow among populations, particularly within the Zagros Mountains, where most populations are concentrated. This pattern is consistent with the presence of admixture across a large proportion of populations and suggests that historical or ongoing connectivity has limited genetic divergence.

The widespread admixture observed among populations likely reflects repeated interbreeding among previously differentiated lineages, a process commonly reported in mountain plant species with overlapping distribution ranges and episodic connectivity (Abbott et al. 2013; Goulet et al. 2016; Mallet 2005; Shriner 2023). In *N. glomerulosa*, such admixture may have been facilitated by past climatic oscillations that periodically expanded suitable habitats, allowing contact among populations that are currently geographically separated.

The dominance of Zagros populations within multiple genetic clusters supports the role of the Zagros Mountains as a major corridor of genetic exchange linking the Alborz and Yazd–Kerman regions. The complex topography and heterogeneous habitats of the Zagros Mountains may promote both local differentiation and recurrent gene flow, resulting in the observed mosaic genetic structure rather than clear phylogeographic breaks.

### 4.3 The Influence of Environmental Factors

Environmental heterogeneity appears to play an important role in shaping genetic differentiation among *N. glomerulosa* populations. Altitude influences temperature and precipitation patterns, with precipitation being more influential at lower altitudes and temperature becoming critical at higher altitudes (Karami et al. 2022). These variations likely drive adaptive divergence among populations across the altitudinal range of 1500–4000 m. Longitude and latitude also contribute to genetic differentiation. Populations MB, ZGD, and TMK, located at higher latitudes, are isolated from others (Figure 6). This suggests that geographic distance may act as a barrier to gene flow, leading to greater genetic distance with increasing longitude or latitude. Variations in altitude, slope, and aspect can create microhabitat discontinuities that restrict pollen and seed dispersal, leading to partial isolation even over short geographic distances (Degen et al. 2006; Rousset 1999; Torroba‐Balmori et al. 2017). Such topographic barriers are especially effective in mountainous regions and may explain localized differentiation despite overall weak regional structure (Han et al. 2025). Similar patterns have been reported in other montane species, where environmental gradients interact with geography to produce fine‐scale genetic structuring without complete isolation (McGreevy Jr. et al. 2024; Morente‐López et al. 2018; Wang and Bradburd 2014). In *N. glomerulosa*, this suggests that genetic structure is driven not by strict geographic separation, but by an interaction between environmental filtering and intermittent gene flow.

### 4.4 Conservation of *N. glomerulosa*

During field excursions conducted for population sampling, we made repeated qualitative observations indicating that several populations of *N. glomerulosa* are under significant anthropogenic pressure. These observations, although not quantified as part of the analytical dataset, consistently revealed the impacts of overgrazing, habitat loss associated with urban expansion, and uncontrolled harvesting for medicinal use.

The vulnerability of this species is exacerbated by its small population sizes and restricted distribution range. Mountainous regions, in particular, appear to be disproportionately affected, as rapid urbanization and increasing human activity in these areas coincide with habitat degradation and intensified harvesting pressure.

Based on these field‐based observations, conservation efforts should prioritize the protection of mountainous habitats where *N. glomerulosa* populations are most exposed to disturbance. Recommended strategies include the regulation of harvesting practices, the establishment of protected areas, and the development of ex‐situ conservation measures such as seed banking. Long‐term conservation will require coordinated actions involving local communities, policymakers, and researchers to safeguard this ecologically and medicinally important species.

## 5 Conclusions

This study offers a thorough evaluation of the genetic diversity and population structure of *Nepeta glomerulosa*, a semi‐endemic medicinal plant found in the Irano‐Turanian region. We conducted an analysis using SRAP markers on 188 accessions from 18 populations located in the major mountain ranges of Iran, where the species is distributed: Alborz, Zagros, and Khorassan‐Kopet Dagh. The species exhibits moderate genetic diversity (He = 0.2605), with the Alborz range showing the highest diversity, suggesting it could be the center of origin. Nonetheless, this diversity falls below the average for short‐lived perennials, prompting concerns regarding conservation. Three distinct genetic clusters were identified, corresponding to geographic regions, with significant admixture observed in nearly half of the samples, indicating complex gene flow and population differentiation. Geographic and environmental factors, such as altitude, edaphic conditions, and geographic distance, play a crucial role in shaping genetic structure. Given the species' restricted range, low population numbers, and the risks posed by overgrazing and habitat degradation, immediate conservation actions are essential, especially in sensitive mountainous areas. Analysis of historical links indicates an origin in the Alborz region, offering valuable insights into its evolutionary history.

The results establish a foundation for successful conservation approaches for *N. glomerulosa* and underscore the importance of integrating genetic and environmental factors into strategic planning. Future investigations with broader sampling, particularly from Afghanistan, alongside the application of next‐generation sequencing technologies, could significantly deepen the comprehension of this medicinally significant species. Prompt conservation measures, integrating on‐site protection, sustainable harvesting practices, and off‐site initiatives, are crucial for ensuring its enduring survival. Collaboration among local communities, policymakers, and experts will be essential for successful implementation.

## Author Contributions

**Sahar Karami:** conceptualization (equal), data curation (equal), formal analysis (equal), methodology (equal), writing – original draft (equal). **Hamid Ejtehadi:** conceptualization (equal), data curation (equal), funding acquisition (equal), project administration (equal), writing – review and editing (equal). **Jamil Vaezi:** data curation (equal), writing – review and editing (equal). **Hamid Moazzeni:** conceptualization (equal), data curation (equal), funding acquisition (equal), project administration (equal), supervision (equal), writing – review and editing (equal).

## Conflicts of Interest

The authors declare no conflicts of interest.

## Supporting information


**Data S1:** Morphometric measurements of five key traits in *Nepeta glomerulosa*. This dataset includes morphological measurements for five main characters: plant height (ph), bract length (bl), calyx length (cl), corolla length (ctl), and bract color (bc), recorded from 134 individuals across 18 populations. These traits were used to assess morphological variation and its relationship with genetic diversity and environmental factors.

**Data S2:** Environmental characteristics of Nepeta glomerulosa populations across Iran. This dataset includes geographic coordinates (longitude and latitude), elevation (altitude), topographic features (slope and aspect), and soil properties (nitrogen—N, potassium—K, phosphorus—P, carbon—C, calcium—Ca^2+^, pH, and electrical conductivity—EC) collected from various sampling sites across the species' distribution range in Iran.

**Appendix S1:** ece373294‐sup‐0003‐FiguresS1‐S4.docx.
**Figure S1:** A sharp peak with the highest DK at K = 3 using STRUCTURE.
**Figure S2:** The heat map reveals how SRAP loci are correlated and differentiated in various populations.
**Figure S3:** Principal component analysis (PCA) for 18 populations of N. glomerulosa based on genetic data.
**Figure S4:** The influence of traits on the separation of subspecies along the first and second axes. Influence of traits; a = based on the first and second axes; b = based on the first axis; c = based on the second axis.

## Data Availability

The authors confirm that the data supporting the findings of this study are available within the article [and/or] its Supporting Information.
