# Supplementary material for: Genetic Diversity, Population Structure, and Environmental Influences in Nepeta glomerulosa : Implications for Conservation in the Irano‐Turanian Region
Source: Ecol Evol. 2026 Apr 29;16(5):e73294. doi: 10.1002/ece3.73294 (PMC13127229; doi:10.1002/ece3.73294)
Supplement: Supplementary file 3 — Appendix S1: ece373294‐sup‐0003‐FiguresS1‐S4.docx. Figure S1: A sharp peak with the highest DK at K = 3 using STRUCTURE. Figure S2: The heat map reveals how SRAP loci are correlated and differentiated in various populations. Figure S3: Principal component analysis (PCA) for 18 populations of N. glomerulosa based on genetic data. Figure S4: The influence of traits on the separation of subspecies along the first and second axes. Influence of traits; a = based on the first and second axes; b = based on the first axis; c = based on the second axis. [file ECE3-16-e73294-s001.docx]

**Assessment of genetic diversity and structure of the semi-endemic medicinal plant of *Nepeta***

***glomerulosa*, using SRAP markers**

Sahar Karami^a^, Hamid Ejtehadi^a^, Jamil Vaezi^b^, Hamid Moazzeni^b,^ *

^a^ Quantitative Plant Ecology and Biodiversity Research Lab, Department of Biology, Faculty of Science, Ferdowsi University of Mashhad, Mashhad, Iran; [saharkarami238@gmail.com](mailto:saharkarami238@gmail.com) (S.K); [hejtehadi@um.ac.ir](mailto:hejtehadi@um.ac.ir) (H.E);

^b^ FUMH Herbarium, Department of Biology, Faculty of Science, Ferdowsi University of Mashhad, Mashhad, Iran; ; [jvaezi@yahoo.com](mailto:jvaezi@yahoo.com) (J.V); [hmoazzeni@um.ac.ir](mailto:hmoazzeni@um.ac.ir) (H.M)

^*^ Corresponding author: [hmoazzeni@um.ac.ir](mailto:hmoazzeni@um.ac.ir)


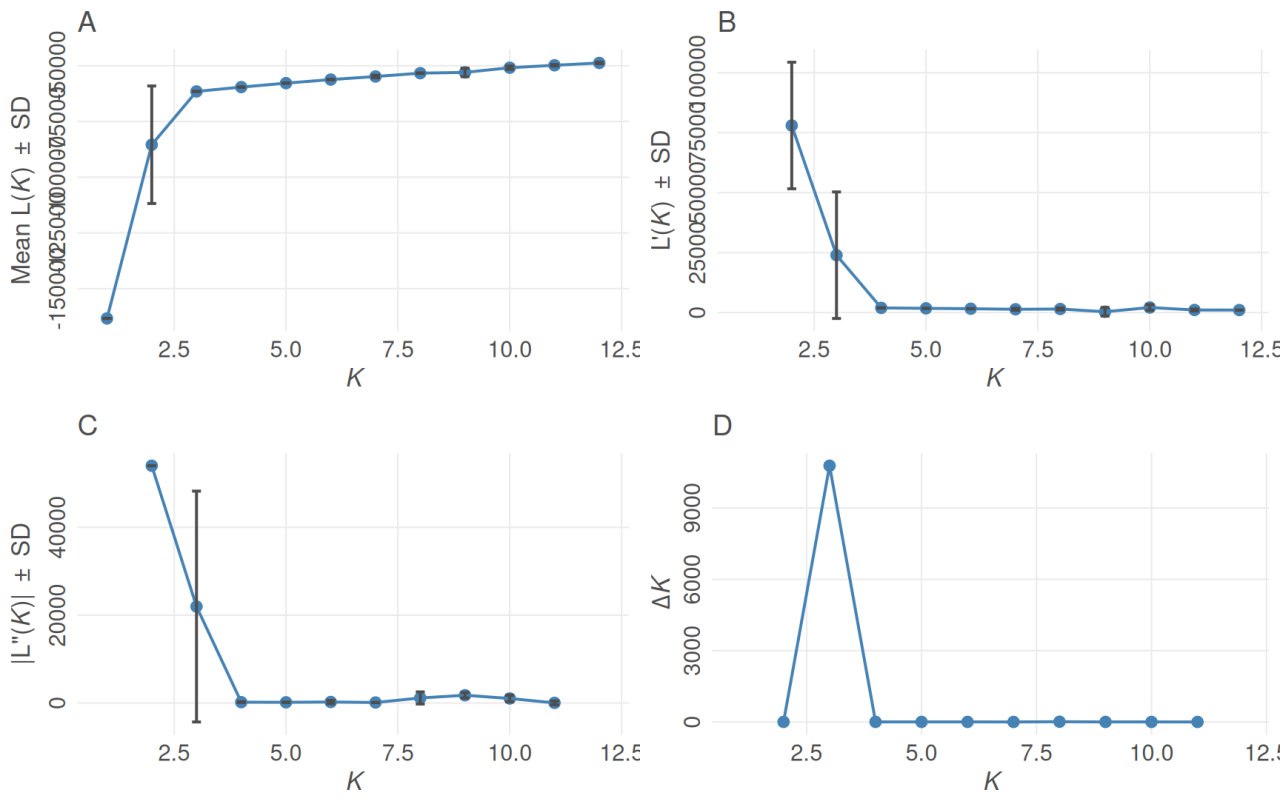


**Fig. 1S.** A sharp peak with the highest DK at K = 3 using STRUCTURE


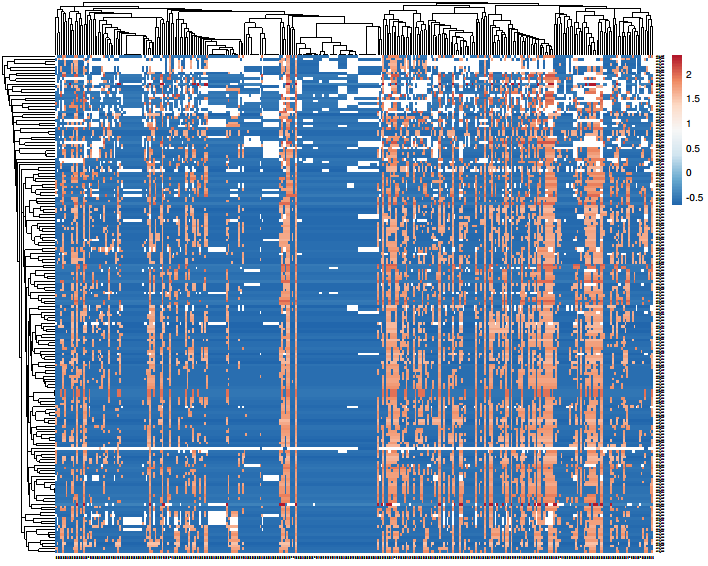


**Fig. 2S.** The heat map reveals how SRAP loci are correlated and differentiated in various populations


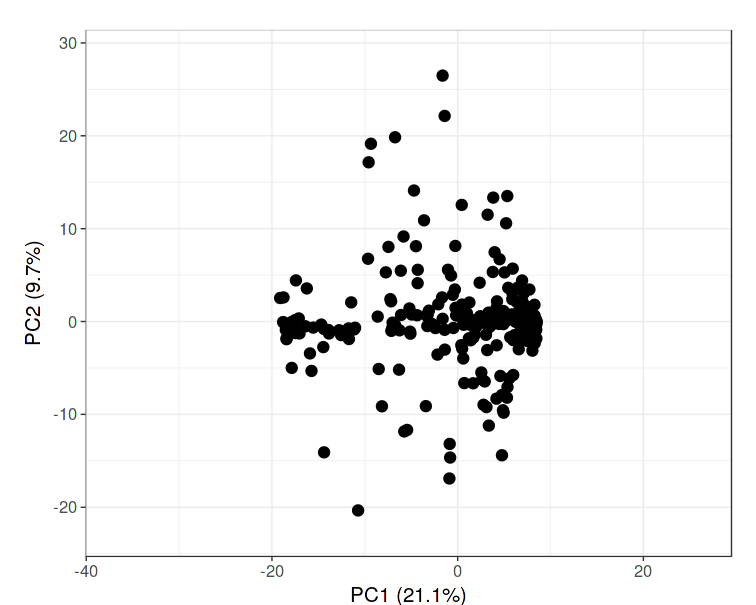


**Fig. 3S.** Principal component analysis (PCA) for 18 populations of *N. glomerulosa based on genetic data*


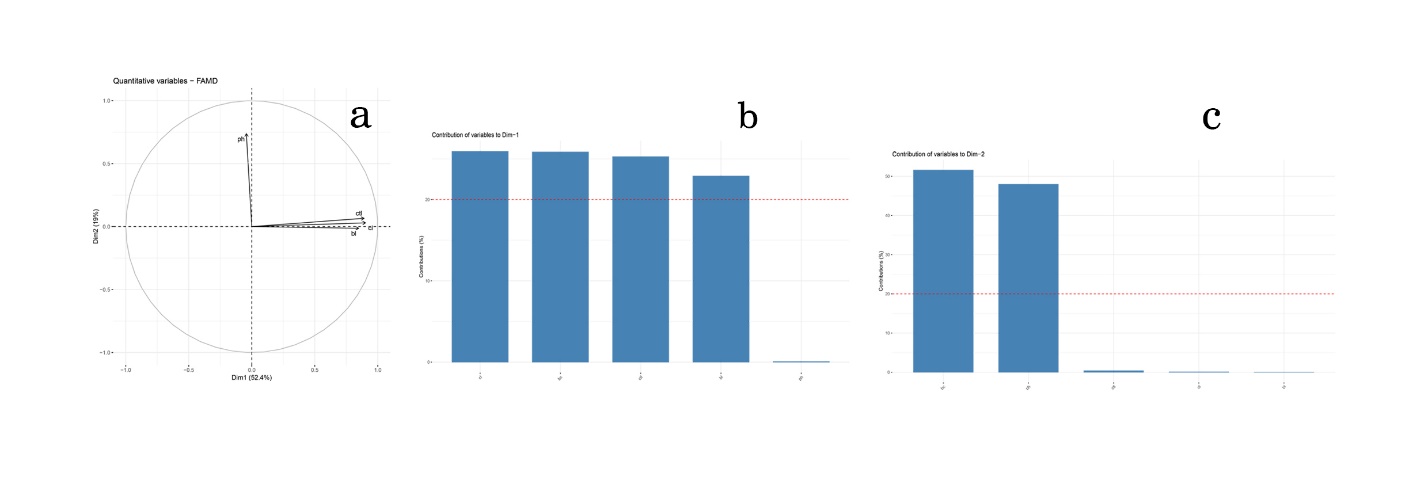


**Fig. 4S.** The influence of traits on the separation of subspecies along the first and second axes. Influence of traits; a= based on the first and second axes; b= based on the first axis; c= based on the second axis
